# Long term outcome of Tenon’s patch graft in corneal perforation secondary to neurotrophic keratitis: A case report on a 4-year anatomical functional outcome

**DOI:** 10.1016/j.ijscr.2021.106046

**Published:** 2021-05-31

**Authors:** Simmy Chaudhary, Sayan Basu, Pragnya R. Donthineni

**Affiliations:** a The Cornea Institute, L V Prasad Eye Institute, Hyderabad, India; b Center for Ocular Regeneration (CORE), L V Prasad Eye Institute, Hyderabad, India

**Keywords:** Tenon’s patch graft, Corneal perforation, Neurotrophic keratitis, Herpes zoster ophthalmicus, Anterior segment optical coherence tomography, Case report

## Abstract

**Introduction:**

This report describes the long-term outcomes of Tenon’s patch graft (TPG) in a corneal perforation secondary to neurotrophic keratitis and outcome of subsequent successful cataract surgery.

**Presentation of the case:**

A 60-year gentleman presented with a corneal perforation secondary to Herpes Zoster ophthalmicus (HZO). After multiple unsuccessful attempts of cyanoacrylate tissue adhesive application over the perforation, he was referred for a corneal patch graft. Following TPG, he had a tectonically stable cornea that was managed with topical steroids and prophylactic oral Acyclovir. Sequential imaging of the cornea using high-resolution anterior segment- optical coherence tomography (HR-ASOCT) was done to monitor wound healing. Fifteen months later, he underwent uneventful cataract surgery with best-corrected visual acuity improving to 20/30 at 1-month.

**Discussion:**

Serial imaging of the site of perforation with HR-ASOCT revealed that a fluffy, oedematous TPG in the early postoperative period transitioned into a hyper-reflective, thin, and compact graft over 3-4 months. Despite the corneal thickness at the site of perforation being only 142 μm, the wound had adequate tensile strength to withstand the altered anterior chamber dynamics during phacoemulsification. The resultant translucent nature of the scar provided superior media clarity and better visual outcomes.

**Conclusion:**

This case demonstrates the efficacy of TPG in acute phase management of corneal perforation following HZO thereby restoring the tensile strength of the cornea, enabling it to withstand the stress of future surgeries like phacoemulsification.

## Highlights

• Tenon’s patch graft is an effective technique in managing corneal perforations.
• It is both easily accessible and economical.
• Resultant thin scar has adequate strength to withstand intraocular procedures.

## 1 Introduction

Herpes Zoster ophthalmicus (HZO) occurs following reactivation of dormant Human Herpesvirus type 3 or Varicella-Zoster virus (VZV), in the ophthalmic division of trigeminal nerve (V1)[1]. It represents 10-20% of total cases of Herpes zoster with incidence and severity increasing with age due to declining cell-mediated immunity [2]. Apart from an acute painful stage, HZO can lead to numerous sight-threatening sequelae like neurotrophic keratopathy, keratitis and cicatrizing lid malpositions that can cause corneal perforations [1,3]. Managing corneal perforation in these eyes is quite challenging, owing to the neurotrophic nature of the cornea. Tenon’s patch graft (TPG) is a technique that has been successfully used in managing large corneal perforations [4,5]. However, there is no literature on its use in the acute management of HZO. Also, no literature exists on long-term serial imaging of TPG and outcomes of subsequent cataract surgery in these eyes. Thus, we report a 4-year anatomical and functional outcome of TPG in treating a corneal perforation in the acute phase of HZO and outcome of subsequent cataract surgery. Serial high-resolution anterior segment optical coherence tomography (HR-ASOCT) was used to study the pattern of wound healing at the site of perforation following TPG. This work has been reported in line with the SCARE criteria [6].

## 2 Presentation of case

**Fig. 1:**
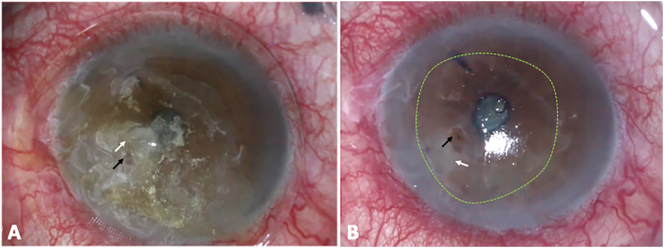
Clinical photomicrographs of the affected eye prior to surgery: A) Preoperative image of the affected eye showing Cyanoacrylate tissue adhesive (white arrow) with an underlying corneal perforation (black arrow) B) Intra-operative image showing the paracentral corneal peroration (black arrow) after removal of the overlying tissue adhesive with a sterile corneal infiltrate (white arrow) and a large epithelial defect (dotted green circle).

A 60-year gentleman visited our hospital with complaints of pain, watering and diminished vision in the left eye for 1.5 months. He was initially diagnosed with HZO and treated with tapering dose of topical steroids, antibiotics, and oral antiviral agents (Valacyclovir 400 mg twice daily) elsewhere. He developed a corneal perforation in due course and following two unsuccessful attempts of TA application was referred to our hospital for a corneal patch graft. At presentation, the affected eye had a best-corrected visual acuity (BCVA) of counting fingers close to face (CF-CF). External examination revealed resolving vesicular lesions over the dermatomal distribution of V1 cranial nerve. Slit-lamp examination (SLE) revealed TA and bandage contact lens (BCL) over a paracentral corneal perforation (Fig. 1). An active leak was noted under the TA with a shallow anterior chamber (AC), hypotonous globe and further details were obscured by TA.

A diagnosis of a corneal perforation (3 ∗ 2 mm) secondary to HZO was made and our patient was taken up for a TPG following another unsuccessful attempt of TA application. TPG was preferred over a corneal patch graft owing to the neurotrophic nature of the cornea. An oversized Tenon’s graft of 4 ∗ 4 mm was harvested from the superior quadrant and plugged into the perforation and secured with fibrin glue (Tisseel Kit, Baxter AG, Vienna, Austria) and overlay sutures using 10-0 monofilament nylon. A small single layer of fresh human amniotic membrane (hAM) was placed over the TPG and AC was formed with air followed by balanced salt solution. After excluding the presence of any leak, another360° overlay hAM was placed over the large corneal epithelial defect. Medial and lateral para-median permanent tarsorrhaphy was performed with 6-0 Polyglactin sutures. Fig. 1 includes the preoperative and intraoperative photographs of the affected eye.

**Fig. 2:**
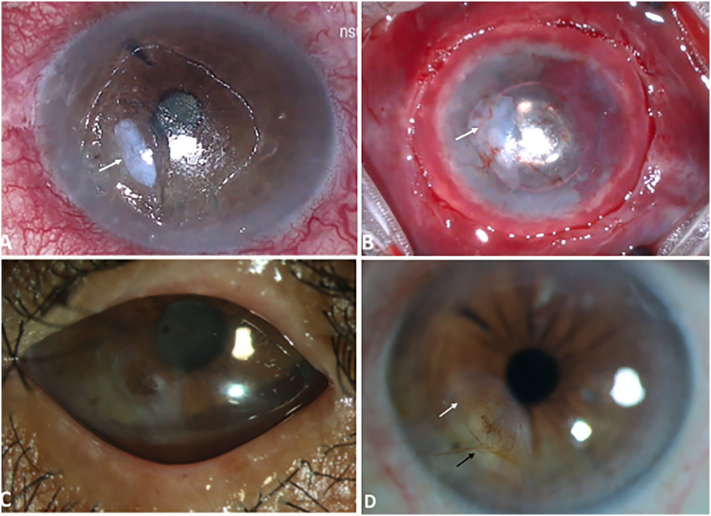
Photomicrographs of the affected eye intra-operative and postoperatively. A) Intraoperative image showing the whitish appearance (white arrow) of the Tenon’s patch graft (TPG) secured over the corneal perforation with fibrin glue. B) Intraoperative image at the end of procedure showing human amniotic membrane (hAM) over the TPG (white arrow). C) Postoperative image at 3 months following TPG, showing complete epithelialization with corneal scarring and intact median and lateral tarsorrhaphy. D) Postoperative image at 32 months post TPG showing corneal scarring (white arrow) and superficial vascularization (black arrow).

Postoperatively, he was prescribed prednisolone acetate 1% eye drops six times daily, moxifloxacin 0.5% eye drops four times daily and topical lubricants in the affected eye and prophylactic dose of oral Acyclovir (800 mg) twice daily. Post-surgery, the patient was followed up on day 1, 2, 2 weeks, 3 months, and six-monthly thereafter for four years. At 3 months follow-up, the BCVA in the affected eye was 20/80 (Log MAR: 0.6). SLE revealed a reduction in the volume of the TPG with complete epithelization of the corneal epithelial defect. Prophylactic dose of oral Acyclovir 800 mg twice daily and lubricating eye drops were continued. At 15 months follow up, the TPG had integrated into the host cornea with translucent corneal scarring, thinning and mild superficial vascularization. However, the patient had diminished vision due to progression of senile cataract with BCVA of CF at 1 m. Oral Acyclovir prophylaxis was continued for 22 months after the acute attack and no recurrence was noted after cessation of the drug. He underwent an uneventful phacoemulsification and intraocular lens implantation in the affected eye 10 months after discontinuing oral antiviral prophylaxis which was not initiated either pre or post-surgery. Fig. 2 depicts the clinical images at follow-up visits. On postoperative day 1, BCVA improved to 20/80 (LogMAR: 0.6) with a tectonically stable cornea. Tapering dose of prednisolone 1% eye drops was started under cover of prophylactic oral Acyclovir. Subsequently, his BCVA improved to 20/30 (Log MAR: 0.17) at 1-month post-surgery.

**Fig. 3:**
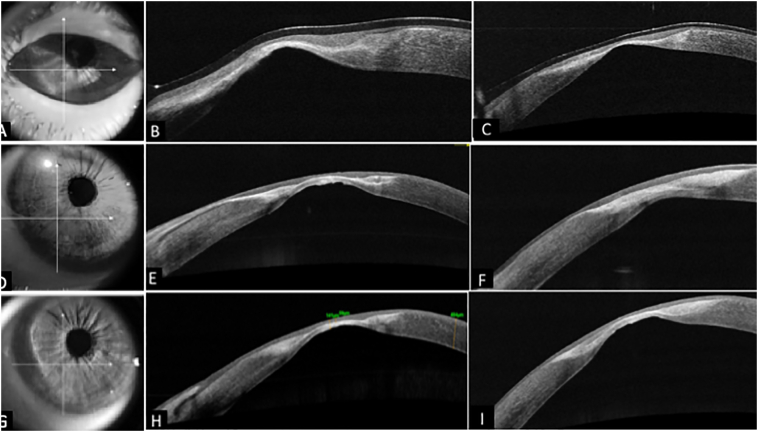
Tissue remodeling of corneal surface post successful Tenon’s patch graft (TPG) over a long-term follow up of three years using serial High-Resolution Anterior segment-OCT (HR-ASOCT) images. HR-ASOCT images (A, B, C) in the top row depict a hyper-reflective, thick TPG with complete epithelization of the corneal surface at 3 months post TPG. D), E), F) HR-OCT at 22 months shows a thin hyper-reflective TPG with regularized corneal surface and pseudo cornea formation posteriorly. G), H), I) at 32 months with well integrated TPG with the thinnest pachymetry of 142 μm at the site of perforation.

Serial HR- ASOCT (Optovue, Fremont, CA) was performed to monitor wound healing at the site of perforation (Fig. 3). The TPG had a swollen appearance due to imbibition of aqueous from AC at 1 week post-surgery with ongoing epithelization, which completed by 2 weeks. Over next 3 months, the hAM disintegrated and TPG underwent deturgescence to become thinner. Corneal epithelization occurred with a regular anterior surface and a pseudo cornea formation posteriorly. Over 5–6 months following surgery, the TPG became thinner, compact, translucent due to tissue remodeling with a corneal thickness of 142 μm at the site of perforation.

## 3 Discussion

Numerous surgical techniques have been described to manage corneal perforations with successful anatomical wound closure. Cyanoacrylate TA is one such technique that is inexpensive, accessible and effective in managing small perforations [7]. But, it does not add volume to the site of perforation and is ineffectual in managing large or peripherally located perforations. Multi-layered hAM and corneal patch grafts act as volume replacing techniques in managing large perforations but are limited by cost, inaccessibility to hAM and dearth of donor corneas [[8], [9], [10]]. Conjunctival advancement pedicles/flaps also aid in managing impending perforations. However, they promote healing with undesirable corneal vascularization [11]. TPG, on the other hand, is inexpensive, easily accessible and effective in sealing large perforations up to 6 mm in diameter [11]. It also eliminates the risk of disease transmission and dependency on eye banks. TPG harbors autologous fibroblasts that aid in its incorporation into host cornea without causing extensive scarring [12]. Though TPG attains successful wound closure, it does not add volume and results in corneal thinning at the site of perforation. There is no report in the existing literature on the long-term outcomes of TPG demonstrating the wound’s ability to withstand the stress of anterior segment surgeries.

Our case demonstrates successful closure of a corneal perforation secondary to HZO sequelae. Serial HR- ASOCT imaging of the site of perforation showed that a fluffy and oedematous TPG noted in the early postoperative period, transitioned into a hyper-reflective, thin and compact graft over a period of 3–4 months post-procedure. It integrated well into the host cornea and served as a good scaffold for the epithelium to grow over it, resulting in a regular anterior corneal surface. The intraocular pressure (IOP) is reported to rise to about 40-60 mmHg during a standard coaxial or bimanual phacoemulsification procedure [13,14]. Despite a thickness of only 142 μm at the site of perforation in our case, the wound had adequate tensile strength to withstand altered anterior chamber dynamics and elevated IOP during phacoemulsification. The translucent nature of the scar provided superior media clarity for intraocular manipulations during surgery. Preoperative corneal topography can help assess irregular astigmatism, biometry and planning surgical incisions and further improve outcomes of cataract surgery.

## 4 Conclusion

This case demonstrates the anatomical and functional outcomes of TPG with HR-ASOCT showing the process of wound healing and integration of tissue into the host cornea. Despite the resultant thin scar at the site of perforation, TPG has the tensile strength to withstand stressful interventions like phacoemulsification.

## Funding

10.13039/501100005809Hyderabad Eye Research Foundation, Hyderabad, India.

## Consent

An informed and written consent was obtained from the patient.

## Patient’s perspective

“I was stressed due to repeated failed applications of glue and referral for emergency corneal transplantation with poor expected outcomes. But once the Tenon’s graft procedure was done, I was relieved, happy and confident to undergo cataract surgery later which improved my vision further.

## Declaration of competing interest

None.
